# Establishment and validation of the scoring system for preoperative prediction of central lymph node metastasis in papillary thyroid carcinoma

**DOI:** 10.1038/s41598-018-24668-6

**Published:** 2018-05-03

**Authors:** Wen Liu, Ruochuan Cheng, Yunhai Ma, Dan Wang, Yanjun Su, Chang Diao, Jianming Zhang, Jun Qian, Jin Liu

**Affiliations:** 1 Department of Thyroid Surgery, The First Affiliated Hospital of Kunming Medical University, Kunming, China; 2 Department of health management center, The First Affiliated Hospital of Kunming Medical University, Kunming, China

## Abstract

Early preoperative diagnosis of central lymph node metastasis (CNM) is crucial to improve survival rates among patients with papillary thyroid carcinoma (PTC). Here, we analyzed clinical data from 2862 PTC patients and developed a scoring system using multivariable logistic regression and testified by the validation group. The predictive diagnostic effectiveness of the scoring system was evaluated based on consistency, discrimination ability, and accuracy. The scoring system considered seven variables: gender, age, tumor size, microcalcification, resistance index >0.7, multiple nodular lesions, and extrathyroid extension. The area under the receiver operating characteristic curve (AUC) was 0.742, indicating a good discrimination. Using 5 points as a diagnostic threshold, the validation results for validation group had an AUC of 0.758, indicating good discrimination and consistency in the scoring system. The sensitivity of this predictive model for preoperative diagnosis of CNM was 4 times higher than a direct ultrasound diagnosis. These data indicate that the CNM prediction model would improve preoperative diagnostic sensitivity for CNM in patients with papillary thyroid carcinoma.

## Introduction

Papillary thyroid carcinoma (PTC) is the most common type of thyroid cancer^1–5^. Patients with thyroid nodules often require ultrasound (US) examination^6–8^, which has become the preferred diagnostic method for PTC because of its convenience, sensitivity, non-invasive and non-radioactive nature, and economical and diagnostic value^9^. Cervical lymph node metastasis is the most common metastatic pathway of PTC, with a central metastasis rate of 20–50%^10–12^. However, the sensitivity of US detection of lymph node metastasis is only 20–38%^13–15^. Especially the assessment of deep lymph nodes in central regions is difficult (e.g., posterior pharynx and mediastinum). If analyzed by physicians with limited experience in US imaging, the accuracy might be further reduced, even after combining with other imaging methods. This may cause a delay in 20% of the surgical programs, missing the best timing of treatment and affecting prognosis^10,16^. Since diagnosis of PTC metastasis requires highly sensitive diagnostic methods, there is an urgent need to develop reliable prediction methods of PTC metastasis.

Several studies have indicated that clinical characteristics, including age, gender, and multifocal tumor location, might have a predictive value in the central lymph node metastasis (CNM) of PTC^17–20^. In addition, scans of PTC metastatic lymph nodes often show the same ultrasound and pathological manifestations as the scans of primary lesions, such as microcalcifications and blood flow changes. In this study, we reviewed the ultrasound images of patients with preoperative diagnosis of PTC within the last 10 years, and analyzed the relationship between ultrasonographic features of PTC tumors and CNM. We then developed a novel scoring system that should improve the sensitivity of preoperative CNM diagnosis.

## Results

### Basic characteristics of the research subjects

A total of 3801 patients were preoperatively diagnosed with PTC in the Thyroid Center of Kunming Medical University First Affiliated Hospital from January 2007 to June 2016. The diagnosis of PTC was mainly based on US-guided fine-needle aspiration biopsy. Postoperative pathology confirmed that 3553 cases had PTC and 248 cases were misdiagnosed due to other benign diseases, with a PPV of 93.5%. Therefore, a total of 2862 cases were included in the study, among which 1240 cases were CNM positive and 1622 cases were negative with a metastasis rate of 43.2% using postoperative paraffin pathology. However, only 265 cases (sensitivity of 16.8%) were diagnosed with CNM or suspicious metastasis by preoperative US diagnosis. Moreover, 1918 cases (~67%) were in the modeling group, had a median age of 42 years (ranged 9–88), and included 384 males (20.0%) and 1534 females (80.0%); 856 cases (44.6%) were CNM positive. The central lymph node dissection (CND) median was 8 (5, 12) nodes, and the mean metastases number was 1.7 (±3.4). Further, 944 cases were in the validation group (about 33%), had a median age of 43 years (ranging 10–79), and included 165 males (17.5%) and 779 females (82.5%); 384 cases (40.7%) were CNM positive. The CND median was 8 (5, 12) nodes, and the mean metastases number was 1.5 (±2.7). No significant difference was found in demographic profiles between the two groups.

### Establishment and evaluation of prediction model and its scoring system

**Figure 1:**
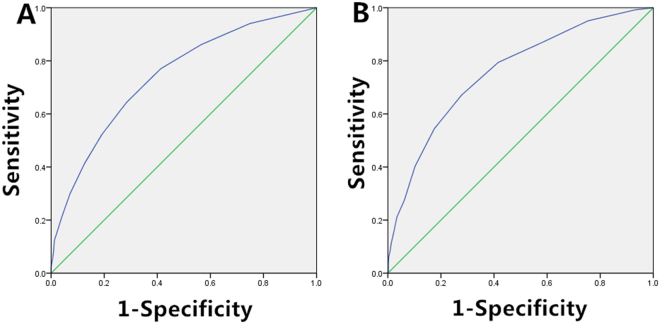
(**A**) The ROC curve of the modeling group. (**B**) The ROC curve of the validation group.

The study was based on clinical features and ultrasonographic characteristics, excluding features that could cause interference. A total of 11 variables were included. The single-factor analysis showed that CNM was associated with gender, age, tumor size, microcalcifications, internal blood supply, RI > 0.7, multiple nodule lesions, and extrathyroid extensions (*P* < 0.05, Table 1). The CNM predictive model and scoring system were established based on the logistic regression model for the diagnosis of patients with PTC. They comprised gender, age, tumor size, microcalcifications, RI >0.7, nodular lesions, and extrathyroid extensions; the *β* value of multiple nodular lesions was the smallest (Table 2). The *β* value of other variables was divided by the corresponding minimum regression coefficient. The prediction scores ranged from 0–9. The proportion of CNM in the subjects increased with increasing scores (Table 3). The results of Hosmer–Lemeshow goodness-of-fit test showed that the logistic regression model had a good calibration (*χ*^2^ = 9.065, *P* = 0.337). The area under the curve (AUC) of ROC was 0.742 [95% confidence interval (CI), 0.720–0.764], indicating a good discrimination of logistic regression model (Fig. 1).

**Table 1:** Relationship between clinical and US characteristics, and CNM of patients in the modeling and validation groups.

Modeling	Total	CNM(−)	CNM(+)	*P* value	Validation Total	CNM(−)	CNM(+)	*P* value
	*N* = 1918	*N* = 1062	*N* = 856		*N* = 944	*N* = 560	*N* = 384	
Gender				<0.001				<0.001
Male	384 (20.0%)	166 (15.6%)	218 (25.5%)		165 (17.2%)	74 (13.2%)	91 (23.7%)	
Female	1534 (80.0%)	896 (84.4%)	638 (74.5%)		779 (82.5%)	486 (86.8%)	293 (76.3%)	
Age				<0.001				<0.001
≤40 years	838 (43.7%)	365 (34.4%)	473 (55.3%)		375 (39.7%)	169 (30.2%)	206 (53.6%)	
>40 years	1080 (56.3%)	697 (65.6%)	383 (44.7%)		569 (60.3%)	391 (69.8%)	178 (46.4%)	
Tumor size				<0.001				<0.001
≤1 cm	1169 (61.0%)	793 (74.7%)	376 (43.9%)		582 (61.7%)	419 (74.8%)	163 (42.4%)	
1–2 cm	530 (27.6%)	211 (19.9%)	319 (37.3%)		273 (28.9%)	118 (21.1%)	155 (40.4%)	
2–3 cm	163 (8.5%)	51 (4.8%)	112 (13.1%)		59 (6.3%)	19 (3.4%)	40 (10.4%)	
>3 cm	56 (2.9%)	7 (0.6%)	19 (5.7%)		30 (3.2%)	4 (0.7%)	26 (6.8%)	
Microcalcification				<0.001				<0.001
No	733 (38.2%)	469 (44.2%)	264 (30.8%)		345 (36.5%)	239 (42.7%)	106 (27.6%)	
Scattered distribution	966 (50.4%)	491 (46.2%)	475 (55.5%)		470 (49.8%)	254 (45.4%)	216 (56.3%)	
Aggregated distribution	219 (11.4%)	102 (9.6%)	117 (13.7%)		129 (13.7%)	67 (12.0%)	62 (16.1%)	
Shape				0.899				0.124
Normal	450 (23.5%)	248 (23.4%)	202 (23.6%)		224 (23.7%)	123 (22.0%)	101 (26.3%)	
Abnormal	1468 (76.5%)	814 (76.6%)	654 (76.4%)		720 (76.3%)	437 (78.0%)	283 (73.7%)	
Internal blood supply				<0.001				0.002
Not rich	1484 (77.4%)	858 (80.8%)	626 (73.1%)		726 (76.9%)	450 (80.4%)	276 (71.9%)	
Rich	434 (22.6%)	204 (19.2%)	230 (26.9%)		218 (23.1%)	110 (19.6%)	108 (18.1%)	
Internal blood vessels				0.737				0.104
Normal	1614 (84.2%)	891 (83.9%)	723 (84.5%)		802 (85.0%)	467 (83.4%)	335 (87.2%)	
Abnormal	304 (15.8%)	171 (16.1%)	133 (15.5%)		142 (15.0%)	93 (16.6%)	49 (12.8%)	
RI > 0.7				<0.001				0.002
No	1494 (77.9%)	878 (82.7%)	616 (72.0%)		746 (79.0%)	462 (82.5%)	284 (74.0%)	
Yes	424 (22.1%)	184 (17.3%)	240 (28.0%)		198 (21.0%)	98 (17.5%)	100 (26.0%)	
Multiple nodular lesions				0.039				0.018
No	730 (38.1%)	426 (40.1%)	304 (35.5%)		342 (36.2%)	220 (39.3%)	122 (31.8%)	
Yes	1188 (61.9%)	636 (59.9%)	552 (64.5%)		602 (63.8%)	340 (60.7%)	262 (68.2%)	
Hashimoto’s disease				0.926				0.001
No	1284 (66.9%)	710 (66.9%)	574 (67.1%)		858 (90.9%)	536 (95.7%)	322 (83.9%)	
Yes	634 (33.1%)	352 (33.1%)	282 (32.9%)		86 (9.1%)	24 (4.3%)	62 (16.1%)	
Extrathyroid extension				<0.001				<0.001
No	1743 (90.9%)	1020 (96.0%)	723 (84.5%)		858 (90.9%)	536 (95.7%)	322 (83.9%)	
Yes	175 (9.1%)	42 (4.0%)	133 (15.5%)		86 (9.1%)	24 (4.3%)	62 (16.1%)	

CNM, central lymph node metastases; RI, resistance index.

**Table 2:** Multivariate logistic regression model and scoring system for CNM prediction.

Parameter	β	SE	Wald	*P*	OR	95% CI for Exp (B)		
						Lower	Upper	Points assigned
Gender								
Female								0
Male	0.53	0.126	17.761	<0.001	1.699	1.327	2.175	2
Age								
>40 years								0
≤40 years	0.763	0.103	55.07	<0.001	2.103	1.753	2.624	2
Tumor size								
≤1 cm			115.202	<0.001				0
1–2 cm	0.963	0.115	70.291	<0.001	2.620	2.092	3.281	3
2–3 cm	1.213	0.19	40.75	<0.001	3.364	2.318	4.882	4
>3 cm	2.385	0.42	32.247	<0.001	10.857	4.767	24.728	9
Microcalcification								
No			16.502	<0.001				0
Scattered distribution	0.395	0.11	12.91	<0.001	1.484	1.197	1.841	1
Aggregated distribution	0.532	0.171	9.738	0.002	1.703	1.219	2.378	2
RI > 0.7								
No								0
Yes	0.293	0.124	5.57	0.018	1.34	1.051	1.709	1
Multiple nodular lesions								
No								0
Yes	0.26	0.105	6.075	0.014	1.297	1.055	1.595	1
extrathyroid extension								
No								0
Yes	1.263	0.198	40.534	<0.001	3.535	2.397	5.215	4
Constant	−1.696	0.129	173.493	0.001	0.183			

CNM, central lymph node metastases; RI, resistance index.

**Table 3:** CNM detection rate of risk score and risk stratification in modeling population and validation population in the established model using preoperative factors.

Risk score	Number	CNM(+)^a^	Risk stratification	Total	CNM(+)^a^
Modeling population				1918 (100.0%)	856 (44.6%)
0	74	12 (16.2%)	Low (0–4)	1067 (55.6%)	306 (28.7%)
1	244	39 (16.0%)			
2	263	68 (25.9%)			
3	240	78 (32.5%)			
4	246	109 (34.3%)			
5	201	103 (51.2%)	High (≥5)	851 (44.4%)	550 (64.6%)
6	162	93 (57.4%)			
7	155	97 (52.6%)			
8	108	75 (69.4%)			
9	68	49 (72.1%)			
10	38	27 (71.1%)			
11	38	35 (92.1%)			
12	29	25 (86.2%)			
13	21	17 (81.0%)			
14	13	11 (84.6%)			
15	3	3 (100.0%)			
16	10	10 (100.0%)			
17	3	3 (100.0%)			
18	1	1 (100.0%)			
19	1	1 (100.0%)			
Validation population				944 (100.0%)	384 (40.7%)
0	41	3 (7.5%)	Low (0–6)	530 (56.1%)	126 (23.8%)
1	116	16 (13.8%)			
2	131	32 (24.4%)			
3	118	28 (23.7%)			
4	124	47 (37.9%)			
5	107	49 (45.8%)	High (≥5)	414 (43.9%)	258 (62.3%)
6	96	55 (57.3%)			
7	70	48 (68.6%)			
8	41	25 (61.0%)			
9	26	20 (76.9%)			
10	24	18 (75.5%)			
11	12	10 (83.3%)			
12	12	9 (75.5%)			
13	12	11 (91.7%)			
14	4	4 (100.0%)			
15	5	5 (100.0%)			
16	3	3 (100.0%)			
17	1	0 (0.0%)			
18	1	1 (0.0%)			

^a^The number of cases with CNM and its risk score or proportion of risk stratification.

The Youden index reached a maximum (0.359) when the score was equal or more than 5 points in the scoring system, and the subjects were divided into a low-risk CNM population group (1067 cases, 55.6%) and a high-risk CNM population group (856 cases, 44.4%) (Table 3). The high-risk group (≥5 points) included 64.3% (550/856) of CNM cases, and its CNM rate (64.6%, 550/851) was significantly higher compared with that of the low-risk population group (<5 points) (28.7%, 307/1067). Under the predicted threshold, the CNM sensitivity, specificity, PPV, NPV, PLR, and NLR were predicated in the scoring system as 64.3%, 71.7%, 64.7%, 71.4%, 2.272, and 0.395, respectively. The detection rates of CNM in the high-risk and low-risk population groups were 64.6% (51.2–100%) and 28.7% (16.2–34.3%), respectively.

The prediction scoring system was then applied to evaluate validation group, showing 43.9% in the high-risk population group (≥5 points) and 56.1% in the low-risk population group (<5 points). The proportion of CNM cases in the two risk stratification groups were 62.3% and 23.8%, respectively, similar to the modeling population. The AUC of ROC was 0.758 (95% CI, 0.727–0.789), which was similar to the discrimination of the modeling population (0.742 in AUC of ROC). The results of Hosmer–Lemeshow goodness-of-fit test showed that the logistic regression model had good consistency (*χ*^2^ = 2.449, *P* = 0.931). Under the predicted threshold, the sensitivity, specificity, PPV, NPV, PLR, and NLR of CNM were predicted as 67.2%, 72.1%, 62.3%, 76.2%, 2.409, and 0.387, respectively, in the scoring system. The detection rates of CNM in the high-risk and low-risk population groups were 62.3% (45.8–100%) and 23.8% (7.3–37.9%), respectively (Fig. 1).

## Discussion

This study has established a prediction model and its scoring system for preoperative diagnosis of CNM in PTC. The model consists of seven variables: gender, age, tumor size, microcalcifications, RI > 0.7, multiple nodular lesions, and extrathyroid extensions. The detection rates of CNM in the modeling and validation groups of high-risk population are 64.6% and 62.3%, respectively. The predictive model has a high prediction consistency, discrimination ability, and accuracy. The same results were obtained using the scoring system, indicating that the model has an important value for CNM risk stratification in patients with PTC, and can improve the sensitivity of preoperative diagnosis of CNM.

Our results indicate that the sonographic features of the primary foci of PTC are related to CNM. Since other primary foci imaging features also correlate with CNM, they might be used in an early CNM diagnosis. Screening of high risk groups for prophylactic central cleaning, and screening of low risk population may improve the diagnostic sensitivity of central lymph node metastasis.

**Figure 2:**
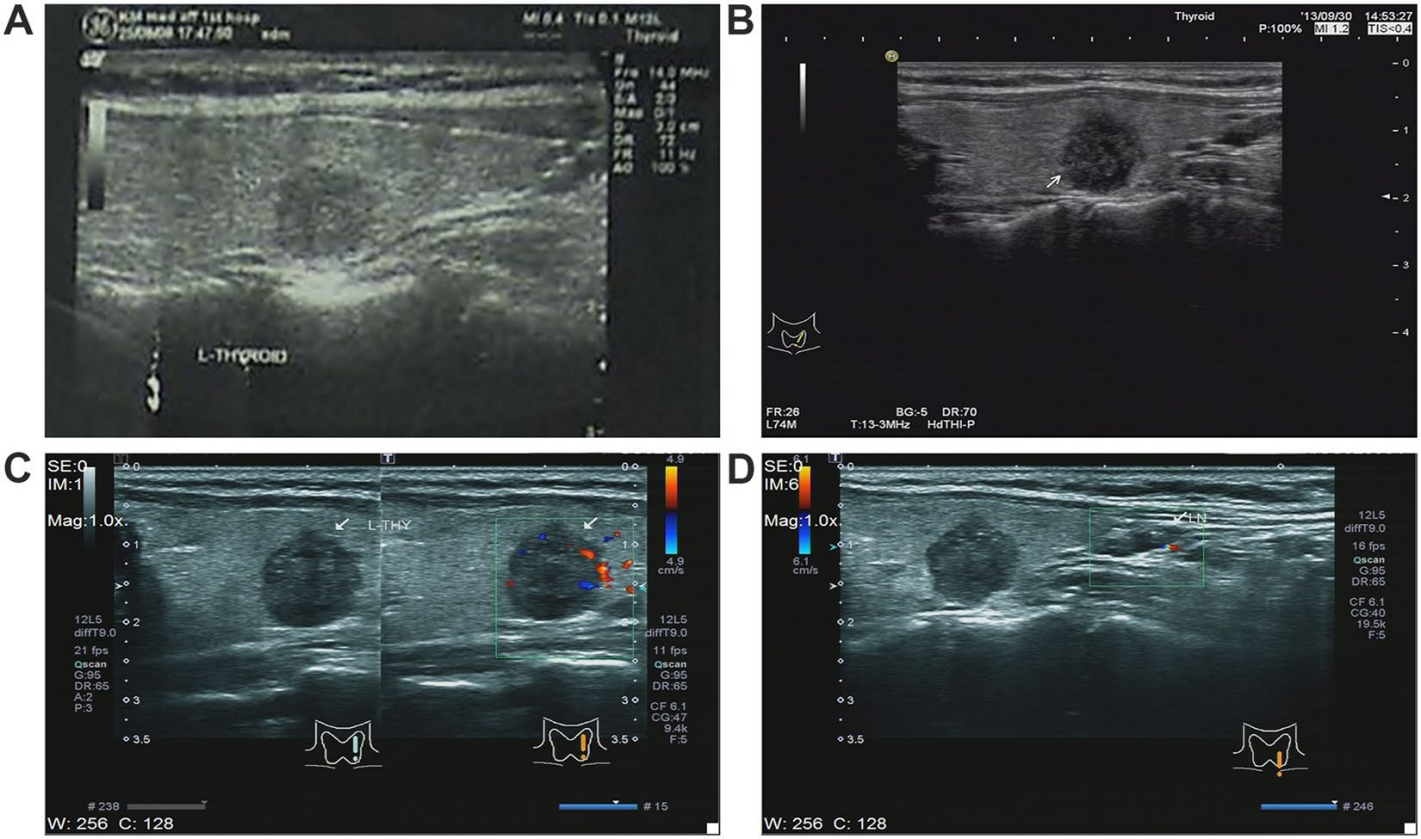
The same left thyroid nodule of a female patient (46 years) was observed for more than 6 years, and changes in the nodule were observed using US images. (**A**) In August 2008, the nodular size found using US was 0.8 × 0.8 cm^2^, with regular shape and without microcalcification; the diagnosis was benign. (**B**) In September 2013, the nodular size was 1.2 × 1.1 cm^2^, with irregular shape and edge angulation, and scattered microcalcification inside the nodule; the diagnosis was suspected malignancy. (**C**,**D**) In September 2014, the nodular size was 1.3 × 1.1 cm^2^, with irregular shape, scattered microcalcification in both the nodule and the left central lymph node medulla; the diagnosis was left thyroid cancer and CNM.

**Figure 3:**
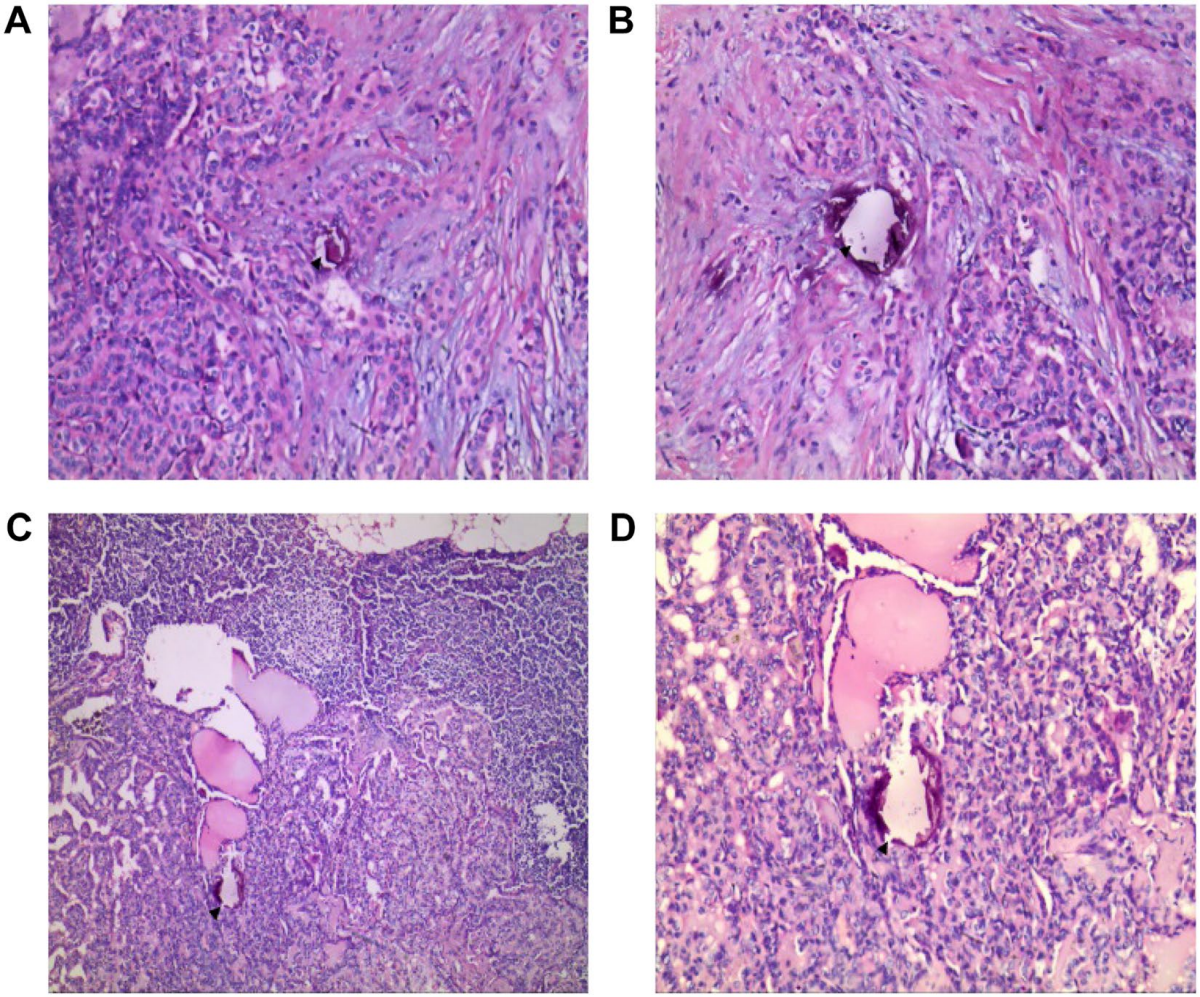
The same patient with postoperative pathological diagnosis of PTC and CNM (2/13). (**A** and **B**) A number of psammoma body formation on the left thyroid foci by H&E staining under 40× magnification and 100× magnification. (**C** and **D**) Visible formation of psammoma bodies inside the nodule by H&E staining under 40× and 100× magnification. H&E, hematoxylin and eosin.

Ultrasonographic microcalcification occurs in about 34–66% of malignant nodules^21–23^, mainly associated with the sand formation of papillary carcinoma. Microcalcification was located in the nipple cadre and fibrous interstitial, and could also be located among solid tumor cell nests; it was extremely rare in other thyroid lesions. Similar structures of psammoma bodies might appear in the cervical lymph node metastases. Concentric roundness-positive body was seen after mucus, calcium, and iron staining, and its center had visible necrotic tumor cells, representing the original place of the formation of psammoma bodies. Our data suggest that psammoma bodies are associated with aggressive behaviors of PTC, and that other features might be used as predictors before the formation of psammoma bodies in lymph node metastases (Figs 2 and 3). A previous study^21^ reported that microcalcification could be used as an independent predictor of CNM in papillary microcarcinoma [odds ratio (OR), 2.378; 95% CI, 1.096–5.158). In this study, 62.3% of the cases showed microcalcifications (1784/2862). Among them, 1185 cases were in the modeling group; the statistical analysis was based on the distribution trend of microcalcification. Our results indicate that both scattered and aggregated distribution of microcalcifications can be used as an independent risk factor for CNM (OR, 1.484; 95% CI, 1.197–1.816 vs OR, 1.703; 95% CI, 1.219–2.378).

**Figure 4:**
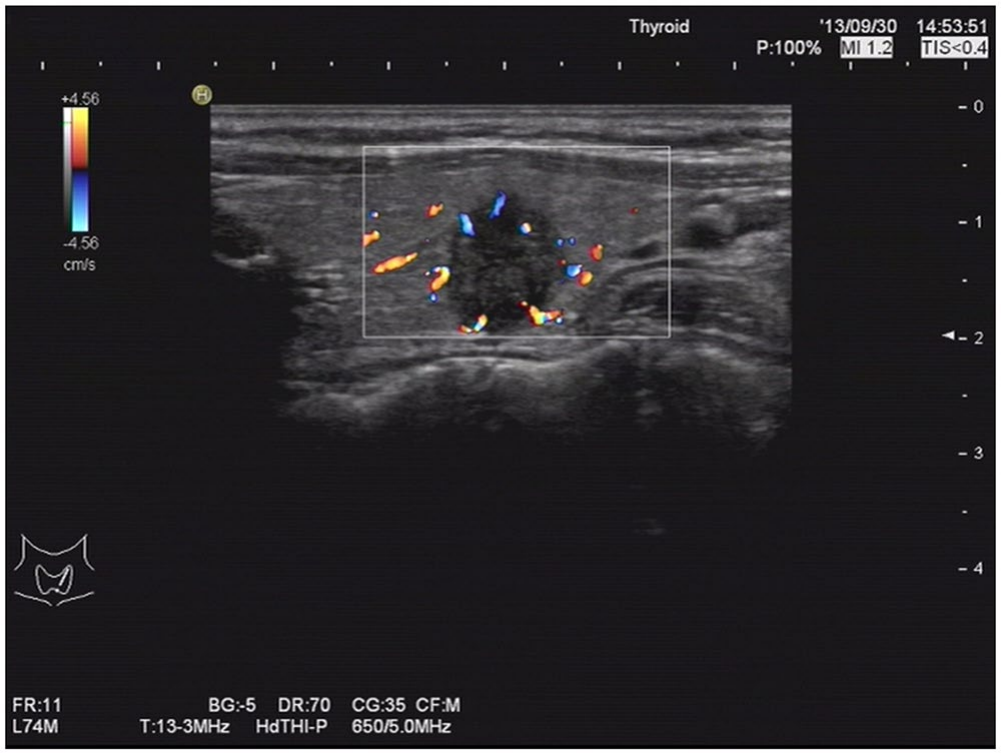
The left thyroid nodular showed increased nodular blood flow signals and blood supply rich and penetration from capsule by Doppler ultrasonography in the same patient.

Several studies have indicated that increased nodular blood flow signals are associated with malignancy^24,25^^.^ However, using a multivariate logistic regression analysis, Moon *et al*.^26^ did not identify an increased nodular blood flow as an independent predictor of thyroid cancer. In this study, only internal blood supply of tumors was used as an indicator, and a total of 1716 cases (~60%) were found to have a visible internal blood supply using Doppler energy. In the modeling group, the internal blood supply of nodules was associated with CNM, and the difference was statistically significant (*P* < 0.001). However, it could not be used as an independent predictor of CNM in the multivariable analysis, so it was not included in this scoring system. Malignant cells can release vascular endothelial growth factor that stimulates angiogenesis within tumors. Adamczewski *et al*.^27^ have suggested that the internal blood supply disorders of thyroid nodules are associated with thyroid cancer. In our study, a total of 304 patients in the modeling group showed an abnormal track of blood vessels or penetration from capsule; this was found irrelevant to CNM using a single-factor analysis (*P* = 0.737) (Fig. 4). These data suggest that the internal tumor blood supply may be important only in PTC, but may not be associated with the lymphatic system metastasis.

Pulse Doppler of normal thyroid arteries presented unidirectional pulsatile spectrum, having a rapid increase in systolic blood pressure that gradually fell to a low-amplitude blood flow in diastolic blood pressure. The RI was typically 0.55–0.66, and increased RI values were commonly seen in PTC and a small amount of nodular goiter cases. The main reason for this was oppression of small blood vessels by hard tumor texture or swollen follicles and hypertrophic fibrous tissue. In this study, RI > 0.7 was set as the threshold, and 622 cases had RI >0.7, accounting for 21.7% of the total cases. Among them, 424 cases were from the modeling group. The multivariable logistic regression analysis showed that RI could be used as an independent risk factor for CNM (OR, 1.388; 95% CI, 1.092–1.763). However, other studies that evaluated the imaging of nodular hardness^21,28^ did not confirm its relationship with CNM, suggesting that other mechanisms might be contributing to the elevated RI values.

The tumor size has been used as a criterion for assessing PTC treatment programs and surgical ranges; larger tumor diameters are associated with cervical lymph node metastases and can increase T staging^29^. Several retrospective analyses with large sample sizes showed that tumor size >1 cm had higher CNM rates^30–34^. An accurate measurement of the tumor size before surgery may help provide a guidance for the diagnosis. In this study, the tumor size was divided into the following four groups: ≤1 cm, 1–2 cm, 2–3 cm, and >3 cm. Single-factor and multivariate analyses showed that the tumor size correlated with CNM (*P* < 0.001); each group could be used as an independent influencing factor and included in the predictive scoring system.

Extrathyroid extension is one of the unfavorable prognostic factors for PTC. Several studies have shown that microscopic extrathyroid extensions have a less adverse effect on the prognosis of PTC patients^35–37^. Moreover, microscopic invasion found in pathological slices may not provide support for timely surgical modification of individual surgical procedures. Therefore, we used intraoperative extrathyroid extension observed by naked eyes as a predictor of one of the variables in the scoring system.

Because PTC often has gland metastases at an early stage, multifocality is one of its significant features. Previous studies have suggested that multifocality could be used as an independent predictor of CNM^38,39^. However, in the specimens with thyroid resection due to benign diseases, 7.3–33.9% of the cases had small foci^40–46^, suggesting that using multifocality may lead to selective bias due to relaxed inclusion criteria. Improvements in US equipment and diagnostic techniques increase the detection rates of thyroid nodules. Liu^47^ *et al*. have suggested that the CLNM was related to multiple regions occupied by tumors in the thyroid but unrelated to multifocality.

Presently, in major hospitals of China, all thyroid nodules of >0.1 cm can be successfully found^8^. This study was strictly based on preoperative US to determine whether multinodular lesions and all nodules (benign and malignant) were included in the analysis, to reduce interventions by human factors as much as possible. The multivariable analysis showed that it could be used as a predictor of CNM and incorporated into the scoring system.

Conventional use of US for CNM diagnosis has a high specificity, but because of low sensitivity and false negative rates, it cannot be used for diagnosis. In this study, the AUC of ROC in the modeling group was 0.742 (95% CI, 0.720–0.764), with a diagnostic sensitivity and specificity of 64.3% and 71.7%, respectively. Validation population results showed that the AUC of ROC was 0.758 (95% CI, 0.727–0.789), with a diagnostic sensitivity and specificity of 67.2% and 72.1%, respectively. In this study, 265 out of 2862 cases were diagnosed with CNM or suspicious metastasis by preoperative US direct diagnosis. The specificity was 96.5%, and the sensitivity was only 16.8%. The sensitivity of this predictive model to preoperative diagnosis of CNM was 3.8 times (64.3% vs. 16.8%) and 4 times (67.2% vs. 16.8%) of direct ultrasound diagnosis.

The CNM detection rate of the scoring system established by preoperative factors was 64.6% and 62.3%, respectively, in the modeling and validation groups of high-risk population. Although the AUC of ROC in the scoring system was 0.742 and 0.758, it was not possible to provide a higher diagnostic specificity. However, in cancer, the sensitivity in metastatic diagnosis is more important than specificity, and a timely diagnosis of patients with CNM is important for the improvement of PTC prognosis.

The predictive indicators included in this study were clinical and US results, and they were easily accessible in the preoperative stage. The US diagnostic indicators were quantified, greatly reducing the interference due to the lack of experience of the US-analyzing physicians and other clinicians. The variables required for the scoring system are the patient’s basic clinical characteristics and routine examination features (US); no invasive operations are needed. The increased sensitivity of preoperative diagnosis can help patients better understand their risk of CNM. Further research in this direction should improve the predictive value of the scoring system.

Although this study was a single-center retrospective study with a large sample size, the diagnosis and treatment processes were standardized and unified. However, some information still could not be accurately collected due to the non-standardized US reports, and human factors could not be completely eliminated. Prospective and multicenter studies are needed to confirm the accuracy of this diagnostic method and improve the scoring system. We also hope that preoperative gene detection and thyroglobulin tests will be supplemented to the scoring system in future research and other scholars’ studies would further improve sensitivity and specificity. Despite the aforementioned shortcomings, this scoring system, as a predictive model for the effective risk stratification of PTC patients using preoperative data, is simple and reliable in a clinical setting, and can be used to improve the diagnostic sensitivity as well as help clinicians in the diagnosis and treatment of PTC.

## Materials and Methods

### Research design

Clinical data of patients with PTC, who were hospitalized from January 2007 to June 2016 in the Thyroid Center, the First Affiliated Hospital of Kunming Medical University, were retrospectively analyzed. After reviewing the tumor US reports and images twice, the relationship between clinical and US characteristics and CNM was analyzed to establish a predictive model and scoring system, which were then validated using validation group. The predictive diagnostic effectiveness of the scoring system was evaluated using consistency, discrimination ability, and accuracy. This study was approved by the ethics committee of the First Affiliated Hospital of Kunming Medical University (2016 Ethical Review L No. 40), and no informed consent was required.

### Research subjects

The patients with PTC enrolled in the Thyroid Center of the First Affiliated Hospital of Kunming Medical University from January 2007 to June 2016 were included in the study. Their PTC conditions were confirmed by postoperative pathology. The inclusion criteria were as follows: initial thyroidectomy, postoperative paraffin pathological diagnosis of PTC, and surgical range including total central lymph node dissection (CND). The US reports were issued by more than two US physicians, and the saved images showed clear signs of major lesions. The exclusion criteria included missing information of medical record, US or thyroid function test, history of head/neck surgery or radiology, history of other malignancy, and presence of other types of thyroid cancer. Moreover, ultrasonic characteristics with the following three situations were not included in the study: (1) interference with other diseases, such as Hashimoto’s disease, could not be excluded because images of US tumor echo in some tumors and surrounding muscle tissues could not be saved at the same time; (2) the composition of tumor and the peripheral blood supply in some US reports were not standardized; and (3) the length/width ratio could not be confirmed by three dimensions in the review of US images. Surgeries were performed by specialists who perform more than 100 surgeries annually^48^. The CND range was according to the cervical lymph node division revised by the American Society of Head and Neck Surgery (2002)^49^ and American Multidisciplinary Consensus (2009)^10^. In our department, the scope of operation includes prophylactic/cure central lymph node (level VI) dissection in preoperative suspicious/diagnosis and confirmation by intraoperative frozen of all PTC patients. The upper, lower, and external bounds of CND were lower edge of hyoid bone, sternal fossa, and medial carotid artery sheath, respectively. The posterior bound was anterior fascia, including all lymph nodes and adipose tissues, paratrachea, pretrachea, and prelarynx.

### Data collection Methods

Age, gender, and other basic information of patients were collected through medical records. US characteristics were collected after reviewing the US reports twice along with the US images. Lesion characteristics of the most important lesions were recorded for multifoci cancer. The size of the maximum cancer nodule was selected in the multifocal carcinomas, and the longest diameter was included in the irregular shape of the carcinoma(s). Two or more nodules found by US were regarded as multiple nodular lesions, including all benign and malignant nodules, except glial nodules. Microcalcification was defined as less than 0.1 cm calcification without acoustic shadow or comet tail sign. Moreover, nodules having coarse calcification and microcalcification simultaneously were also classified as microcalcification^21,24^. According to the distribution trend of microcalcification lesions in tumors, scattered distribution and aggregated distribution were divided. Irregularity or lobulated growth on the edge of tumors was defined as irregular shape^6^. Internal blood supply was divided into rich and not rich based on the number of blood vessels in tumors using Doppler ultrasonography. Internal blood vessels with tortuous and disordered track and penetrating branches were defined as abnormal track. The blood flow resistance index (RI) of tumor was recorded as the highest measured value of all internal blood vessels^50^. The diagnosis of Hashimoto’s disease was confirmed by US as well as by using serum thyroid peroxidase antibodies. All information of US and thyroid function was collected before the last test results (within 1 month before surgery). Extrathyroid extensions included tumors that broken through the capsule, invaded the band muscle, subcutaneous tissues, nerves, or other organs.

### Statistical analysis

Statistical analysis was performed using the SPSS 22.0 statistical software package (IBM, NY, USA). Two-thirds of the cases were randomly assigned into the modeling group and one-third into the validation group. Mean ± standard deviation was used for quantitative data with normal distribution. Skewed distribution used quartile and qualitative data that were expressed as rate and composition ratio. *χ*^2^ test was used for the hypothetical testing data in the modeling group, and variables with *P* < 0.1 and without significant correlation were selected for a multivariable analysis^51^. After applying logistic regression analysis model, a backward stepwise regression was performed. Subsequent variables with *P* < 0.05 were selected, and variables with *P* > 0.1 were excluded according to the results of partial likelihood ratio test. Finally, regression equation and regression coefficients of independent variables were obtained. The selected variables in the logistic regression model were divided by minimum regression coefficient to get a score for each variable in an integer, thereby establishing the predictive scoring system of CNM in PTC. The consistency of risk assessment was assessed using Hosmer–Lemeshow goodness-of-fit test, in which *P* > 0.05 was considered as a good model consistency^50^. The receiver operating characteristic (ROC) curve was used to evaluate discrimination ability of the prediction model and its scoring system. An appropriate cutoff value was selected to calculate the sensitivity, specificity, positive predictive value (PPV), negative predictive value (NPV), positive likelihood ratio (PLR), and negative likelihood ratio (NLR) of the model. A logistic regression analysis in the validation population group was performed using the Enter method to evaluate the diagnostic efficiency of the predictive model (as mentioned earlier) and verify its clinical diagnostic value.
